# Role of CNTN6 in neurodevelopment and neuropathology

**DOI:** 10.3389/fnins.2026.1726979

**Published:** 2026-04-22

**Authors:** Maria M. Shadrina, Anna A. Kashevarova, Yana A. Tyumentceva, Ariuna T. Ranzaeva, Igor N. Lebedev, Marina Y. Khodanovich

**Affiliations:** 1 Laboratory of Neurobiology, Research Institute of Biology and Biophysics, Tomsk State University, Tomsk, Russia; 2 Laboratory of Cytogenetics, Research Institute of Medical Genetics, Tomsk National Research Medical Center of the Russian Academy of Sciences, Tomsk, Russia

**Keywords:** autism, CNTN6, Contactins, genetic variants, mutations, neurite guidance, neurodevelopment, Tourette syndrome

## Abstract

Contactin 6 (CNTN6) is a recently discovered member of the contactins family, which belongs to a group of cell adhesion molecules. This review summarizes the current knowledge about the possible functions of CNTN6 in the organism and its manifestations in animal models and human diseases. Histological, cellular, and molecular studies in rodents have shown the involvement of this protein in neurite guidance, neural network development, and oligodendrocytogenesis. The expression levels in the cerebellum, hippocampus, and visual cortex of rodents vary depending on the period of neurodevelopment. Animal models with a deletion of the *Cntn6* gene have shown impaired spatial orientation and memory patterns. In humans, copy number variation (CNV) analysis and genome-wide association studies (GWAS) found allele-phenotype relationship of this gene with autism spectrum disorder (ASD), intellectual disability (ID), Tourette syndrome (TS), schizophrenia (SCZ), anorexia, and other mental and neurodevelopmental diseases.

## 1 Introduction

Contactins, including contactin 6 (CNTN6), have been intensively studied due to their important role in neurodevelopment and involvement in a number of neuropsychiatric disorders. The contactins family consists of six neuronal cell adhesion molecules, CNTN1 through CNTN6. The contactins are six structurally similar cell adhesion molecules specific to the nervous system and belonging to the immunoglobulin (Ig) superfamily (Stoeckli, 2010). Contactins are expressed primarily in neurons, where they direct neurite outgrowth (Mohebiany et al., 2014), involved in the process of oligodendrogenesis, and the establishment of neuron-glial contacts (Çolako Glu et al., 2014; Zoupi et al., 2018). Despite the structural and functional similarity of contactins, each of them has a special role in the development, restructuring and maintenance of neural networks, as evidenced by differences in the localization and expression levels in the brain throughout life, differences in the phenotype of genetic animal models, and the association of variants of these genes with neuropathologies (Shimoda and Watanabe, 2009; Oguro-Ando et al., 2017).

CNTN6 is the last discovered of the contactins. The role of CNTN6 in the development of the nervous system did not seem critical, since variants of this gene occur in healthy individuals (Huang et al., 2012; Murdoch et al., 2015; Mercati et al., 2016), and *Cntn6*-deficient mice do not exhibit visible phenotypic differences from their wild-type (WT) counterparts (Takeda et al., 2003). However, a number of studies have already shown its participation in establishing a neural network (Zuko et al., 2016a) and oligodendrocytogenesis (Cui et al., 2004), as well as in the development of neuropathologies including autism spectrum disorder (ASD), intellectual disability (ID), Tourette syndrome (TS), schizophrenia (SCZ), and anorexia (Burbach and van der Zwaag, 2009; Kumar and Christian, 2009; Zuko et al., 2013; Kashevarova et al., 2014; Hu et al., 2015; Gandawijaya et al., 2021). These studies indicate *CNTN6* as a candidate gene involved in the complex molecular mechanisms of these pathologies.

In this review, we summarized data regarding the involvement of this protein in the development of pathologies in humans and related them to the results of the studies on animal models and underlying molecular mechanisms. In addition to clinical studies focused on the specific role of contactins in the development of above pathologies, our analysis includes CNTN6-related data from GWAS studies, which are usually contained in the extensive Supplementary material section.

## 2 Brief description of the members of CNTN family: location, expression timeline, and functions

All contactins are glycoproteins with a similar structural organization and are mainly localized in neurons. Their molecular composition includes six N-terminal immunoglobulin (Ig) domains and four fibronectin type III (FNIII) domains. None of the contactins has an intracellular domain. They are attached to the outer side of the cellular membrane using a glycosylphosphatidylinositol anchor (GPI) at the C-terminus (Chatterjee et al., 2019). Contactins interact with transmembrane co-receptors, form cis- or trans-complexes to transmit signals into the cell (Nikolaienko et al., 2016). Co-receptors are transmembrane proteins of the neurexin family (CNTN-associated proteins), L1 family (NrCAM, CHL1), or protein tyrosine phosphatases (PTPRG, PTPRZ) (Nikolaienko et al., 2016; Chatterjee et al., 2019). Table 1 summarizes the literature data on the physiological functions, localization of contactins in the CNS at different stages of neurodevelopment, the phenotype of knockout mice, and the relationship of contactins with diseases in humans. All members of the family play a role in establishing and maintaining connections and interactions between neurons, as well as between neurons and glia, at different stages of neurodevelopment (Chatterjee et al., 2019).

**Table 1:** Key characteristics of contactin family proteins (CNTN1–CNTN6) found in rodents and human brain.

Protein	Object	Location and intensity of expression in development				Knockout mouse phenotype/associated diseases in human	Known physiological functions
		Prenatal	Early postnatal	Adult	Elderly		
CNTN1	Rodents	Most active in spinal cord, medulla oblongata, midbrain, thalamus, and colloid plexus (Massaro et al., 2012; Yoshihara et al., 1995)	Widespread throughout the whole brain (Yoshihara et al., 1995)	Mostly in gray matter: cerebral cortex, caudate putamen, cerebellum, hypothalamus, hippocampus (Massaro et al., 2012; Yoshihara et al., 1995; Shimazaki et al., 1998)	Decreased in the hippocampus, constant in the cerebellum and cerebral cortex in comparison with adulthood (Shimazaki et al., 1998)	Myelin detachments, impaired nerve signaling, died at 18 days after birth (Boyle et al., 2001; Çolako Glu et al., 2014)	Axogenesis, myelination (Falk et al., 2002; Shimoda and Watanabe, 2009), neurogenesis, synaptic plasticity, memory (Puzzo et al., 2015)
	Human	No data	No data	Most active in the cortex and cerebellum (Kamei et al., 2000)	No data	Lethal congenital myopathy (Compton et al., 2008), CIDP (Querol et al., 2013), cancer progression and metastasis (Liang et al., 2020)	
CNTN2	Rodents	Most active in the olfactory bulb, cerebral neocortex, hippocampus, inferior colliculus, and basilar pons (Yoshihara et al., 1995)	Most active in cerebellar granule cells, low in cerebral neocortex, absent in the white matter (Yoshihara et al., 1995)	Most active in cerebellar granule cells, moderate in the olfactory bulb, hippocampus (CA3 field), and pontine nuclei, in white matter (Yoshihara et al., 1995)	No data	Spontaneous seizures, motor, and cognitive impairments (Stogmann et al., 2013; Savvaki eat al., 2008)	Myelination (Savvaki et al., 2008; Zoupi et al., 2018), modulation of neurogenesis (Ma et al., 2008), axonal guidance (Wolman et al., 2008; Fan et al., 2024) Genetic modifier of PIGA-related epilepsy (Thorpe et al., 2025)
	Human	No data	No data	Most active in the cortex, putamen, medulla, corpus callosum and cerebellum; mid-levels in the thalamus, substantia nigra and hippocampus, caudate nucleus and amygdala (Kamei et al., 2000)	No data	Multiple sclerosis (Singh et al., 2015) Alzheimer’s disease (Chatterjee et al., 2018)	
CNTN3	Rodents	Low levels in the whole brain, a little increase in the caudal portion of the dorsal thalamus (Yoshihara et al., 1995)	Similar to adult expression levels (Yoshihara et al., 1995)	Expressed in the cerebellum, hippocampus, olfactory structures, basal ganglia, thalamic and amygdaloid nuclei, and brainstem neurons (Yoshihara et al., 1995)	No data	Viable, no visible differences from WT mice, elevated blood urea nitrogen and circulating triglyceride levels in males, thick ventricular wall in females (Groza et al., 2023)	Neurite outgrowth and guidance (Yoshihara et al., 1995; Hussman et al., 2011)
	Human	Very weak in the frontal lobes (Korotkov et al., 2022)	In the frontal lobes, peak expression occurs in the first year of life when the level of expression decreases (Korotkov et al., 2022)	High levels in the cerebral cortex and amygdala (Kamei et al., 2000). In the frontal lobes, expression decreases with age after 17 years (Korotkov et al., 2022)	Absent in the frontal lobes (Korotkov et al., 2022)	Tuberous sclerosis (Korotkov et al., 2022) ASD (Morrow et al., 2008) Glioblastoma (Zhu et al., 2019)	
CNTN4	Rodents	Low levels in the whole brain, a little increase in the olfactory bulb, the medulla oblongata and the inferior colliculus (Yoshihara et al., 1995)	Significant increase, especially in the cerebral neocortex, inferior colliculus, and cerebellum (Yoshihara et al., 1995)	Similar to postnatal, high levels in cerebral cortex, CA3 zone of hippocampus, cerebellum, thalamus, paracentral gyrus, amygdala, parietal/frontal lobes (Yoshihara et al., 1995)	No data	Impaired morphology of neurons in hippocampus, cortex, visual and olfactory systems (Oguro-Ando et al., 2021; Bamford et al., 2024; Osterhout et al., 2015; Kaneko-Goto et al., 2008). Increased fear conditioning behavior, autism-like behavior (Oguro-Ando et al., 2021; Wang et al., 2025)	Axon growth and guidance, synaptic plasticity, fear behavior (Oguro-Ando et al., 2021; Bamford et al., 2024; Osterhout et al., 2015; Kaneko-Goto et al., 2008)
	Human	No data	No data	High-levels in cerebellum, mid-levels in frontal and occipital lobes, low levels in other brain regions (Kamei et al., 2000)	No data	3p deletion syndrome (Fernandez et al., 2008) Alzheimer disease (Blacker et al., 2003) Schizophrenia (Fromer et al., 2016) ASD (Nava et al., 2014)	
CNTN5	Rodents	Low levels in the whole brain (Ogawa et al., 2001)	Increases in cochlear nucleus, cerebral cortex, olfactory bulbs, facial nerve tract, cerebellum compared to prenatal level (Ogawa et al., 2001)	Overall decrease of expression; most high levels retain in auditory pathway, olfactory structures, thalamus, cortex (layers II-IV), hippocampus, cerebellum, piriform cortex, inferior olive, and facial nucleus (Ogawa et al., 2001)	No data	Decreased alaninaminotransferase level in males (Groza et al., 2023) Reduced number of fibers and synapses in the auditory system Shimoda and Watanabe, 2009) Decreased body fat and weight, increased blood pressure and heart rate, increased metabolism with normal activity in males (Smirnov et al., 2018) Aberrant responses to acoustic stimuli (Li et al., 2003)	Circuit formation and connectivity in early development, synaptic maintenance during adulthood (Shimoda et al., 2012; Kleijer et al., 2015)
	Human	No data	No data	High levels in the amygdala and cerebral cortex, mostly in occipital lobes (Kamei et al., 2000)	No data	ASD (Mercati et al., 2016; Schmilovich et al., 2022)	
CNTN6	Rodents	Low levels in cerebrum and cerebellum (Lee et al., 2000)	High levels in cerebrum, low levels in cerebellum (Lee et al., 2000). High levels in the thalamus, motor cortex, somatosensory cortex and visual cortex (Zuko et al., 2016b)	Similar to postnatal, high level in the thalamus, cerebellum, low levels in the motor cortex, somatosensory cortex and visual cortex (Zuko et al., 2016b)	No data	Reduced parallel fiber synapses, delayed corticospinal development (Sakurai et al., 2009), impaired motor coordination (Sakurai et al., 2009), spatial learning (males) (Mu et al., 2018), reproductive behavior and olfactory function (males) (Zhang et al., 2024)	Synaptogenesis at glutamatergic terminals, axon guidance in postnatal development (Sakurai et al., 2009, 2010) Oligodendrogenesis, neuron differentiation (Quarles, 2007; Zuko et al., 2013) Regulation of apoptosis (Zuko et al., 2016a)
	Human	No data	No data	Maximum expression in cerebellum, mid-low levels through the whole brain (Kamei et al., 2000)	No data	ASD (Mercati et al., 2016), TS (Huang et al., 2017)	

### 2.1 CNTN1

CNTN1 [glycoprotein gp135, F11 in chicken (Ranscht, 1988) and F3 in mice (Gennarini et al., 1989)] was first described in 1988 as “contactin” because no other related proteins were known to exist (Ranscht, 1988). This work also provided a description of the structure of the CNTN1 protein. In humans, CNTN1 was first described in the study by Reid et al. (1994). It was found at synapses and mediates axon-glia interactions during growth through association with extracellular matrix components (tenascin-R, tenascin-C) and RPTPß/phosphatane (Falk et al., 2002; Bizzoca et al., 2009). In addition, CNTN1 modulates brain myelination (Shimoda and Watanabe, 2009): it promotes the formation of paranodal junctions by complexing with the transmembrane protein CNTNAP and organizing axonal subdomains at the nodes of Ranvier for interaction with other Ig-CAMs (Falk et al., 2002; Bizzoca et al., 2009). Mice knocked out of this gene died at 18 days after birth and exhibited myelin detachments and impaired nerve signaling (Boyle et al., 2001; Çolako Glu et al., 2014).

In mice, Cntn1 is most actively expressed during the period of embryonic neural network development (Ranscht, 1988; Massaro et al., 2012), whereas in the adult brain this protein is expressed to a lesser extent and is found only in the hippocampus and cerebellum (Ranscht, 1988; Massaro et al., 2012). Moreover, Puzzo et al. (2015) showed that Cntn1 overexpression improved hippocampal synaptic plasticity and memory in aged mice. In rats, the protein level in the cerebellum and cerebral cortex remains stable throughout life, whereas protein levels in the hippocampus decrease by 30 months (Shimazaki et al., 1998). In humans, the age-related dynamics of CNTN1 expression have not been studied; only in adulthood the localization of expression was investigated (Table 1). According to Kamei et al. (2000), the maximum expression level is observed in the cerebellum while other parts of the brain show medium-low levels of expression.

A case report showed that variants in the *CNTN1* gene play a role in the development of lethal congenital myopathy in humans (Compton et al., 2008). In a case–control study, the serum of the patients with chronic inflammatory demyelinating polyradiculoneuropathy showed immunoprecipitation against CNTN1 with rat cultured hippocampal neurons and paranodal structures (Querol et al., 2013). Additionally, it is associated with invasion, migration, metastasis, and poor prognosis in several cancers originating from organs, such as the lung, stomach, prostate, esophagus, oral cavity (oral squamous cell carcinoma, OSCC), thyroid, liver, and breast (reviewed by Liang et al., 2020).

### 2.2 CNTN2

CNTN2 [TAX-1 in humans (Tsiotra et al., 1993), Tag-1 in rodents (Dodd et al., 1988), or axonin-1 in chicken (Zuellig et al., 1992)] is primarily expressed in the growth cones of axonal extension of neurons (Chatterjee et al., 2019) and in the juxtaparanode (Zou et al., 2017; Kyriakopoulou et al., 2002; Faivre-Sarrailh and Devaux, 2013), where it is required for potassium channel clustering. The structure of this protein in chickens was first described by Zuellig et al. (1992). Recent studies of cryo-EM structure of CNTN2 revealed its possible role in axo-glial contacts and axon guidance (Fan et al., 2024; Zhang et al., 2025). *Cntn2*-deficient mice demonstrate spontaneous seizures, impaired motor and cognitive abilities (Savvaki et al., 2008; Stogmann et al., 2013). Deletion of *CNTN2* causes shortening of the nodes of Ranvier (Savvaki et al., 2008) that point to an association between this protein and demyelinating diseases. This assumption finds some confirmation in clinical studies: an increase in the CNTN2 protein level in the cerebrospinal fluid of children with multiple sclerosis has been shown (Singh et al., 2015). In addition, CNTN2 acts as a ligand for amyloid precursor protein (APP): it inhibits TGFβ2-mediated neuronal cell death via APP (Tachi et al., 2010), affects neurogenesis by binding extracellular APP, triggering Fe65-dependent neurogenesis and modulation of neurogenesis via interaction with *γ*-Secretase-dependent release (Ma et al., 2008). In this regard, it is important that the reduced levels of CNTN2 have been found in the cerebrospinal fluid of patients with Alzheimer’s disease (Chatterjee et al., 2018). Interestingly, comprehensive molecular studies of genetic variants of *CNTN2* showed that it can act as genetic modifier of PIGA-related epilepsy and contribute to reduced penetrance of PIGA variants (Thorpe et al., 2025).

The peak expression of Cntn2 in rodents occurs during the late embryonic and early postnatal stages of development (Wolfer et al., 1994; Yoshihara et al., 1995; Kyriakopoulou et al., 2002), when it directs axonal growth in the brain (Stoeckli et al., 1991; Pedraza et al., 2001; Kyriakopoulou et al., 2002). In the adult rat brain, this protein is expressed in the hippocampus, olfactory bulb, and cerebellar granule cells (Wolfer et al., 1994; Yoshihara et al., 1995). Interestingly, the expression levels are high in the white matter of the adult rat brain but not of the embryonic and early postnatal brains (Yoshihara et al., 1995). In adult human brain, northern blot analysis (Kamei et al., 2000) revealed the highest expression levels in the corpus callosum, substantia nigra, and medulla. Moderately high expression was found in all other areas except the caudate nucleus and amygdala, where expression levels were extremely low.

### 2.3 CNTN3

CNTN3 and its structure were first described in 1994 by Yoshihara et al. (1994) as brain immunoglobulin superfamily protein 1 (BIG-1) and Connelly et al. (1994) as plasmacytoma-associated neuronal glycoprotein (PANG), independently by each research group. This protein plays an important role in late prenatal and early postnatal neurodevelopment (Yoshihara et al., 1995; Korotkov et al., 2022) but exact function of this protein is less clear than for CNTN1 and CNTN2 (Mohebiany et al., 2014). *Cntn3*-knockout mice demonstrate no visible differences compared to WT mice, but they do have cardiac and metabolic problems (Groza et al., 2023).^1^ In mice, it has been shown to form cis- and trans-complexes with protein tyrosine phosphatase receptor type G (Ptprg) in retinal photoreceptor cells, which indicate a role in selective neuronal coupling during visual signaling (Nikolaienko et al., 2016). In rats, the expression is weak in the prenatal brain, increases after birth, and remains high only in specific subsets of neurons in adulthood (see Table 1 for details) (Yoshihara et al., 1994, 1995). In humans, it has been shown to participate in late prenatal and early postnatal corticogenesis (Korotkov et al., 2022). In case–control study, increased levels of this protein have been observed in demyelinating lesions in patients with tuberous sclerosis (Korotkov et al., 2022). One case has also been documented in which *CNTN3* deletion led to ASD (Sutcliffe, 2008). In addition, CNTN3 expression level was shown as prognostic indicator for the two datasets of patients with glioblastoma (Zhu et al., 2019).

### 2.4 CNTN4

CNTN4 [brain immunoglobulin superfamily protein 2 (BIG-2)], its key structural features and role in the nervous system was first described by Yoshihara et al. (1995). In the same work, he proposed to combine contactins 1–4 (BIG-1/PANG, BIG-2, TAG- l/axonin-1, and F3/F11) into a structurally and functionally related subgroup of the immunoglobulin superfamily. CNTN4 is involved in axon guidance and branching through interaction with APP in the accessory optic system (Osterhout et al., 2015). Cntn4, as well as Cntn5 and Cntn6, have also been shown to selectively interact with the tyrosine phosphatase gamma (Ptprg) coreceptor via the carbonic anhydrase domain present in mice but not humans (Bouyain and Watkins, 2010a, 2010b). This interaction is important for the development and maintenance of neural tissues (Mohebiany et al., 2014; Osterhout et al., 2015), in the olfactory system (Kaneko-Goto et al., 2008) in particular. In the adult rat brain, expression is low in prenatal development, significantly increases in many brain structures after birth (see Table 1 for details), and remains high throughout life (Yoshihara et al., 1995). However, a number of animal studies have indicated the important role of CNTN4 in early neurodevelopment (Oguro-Ando et al., 2017, 2021; Zhao et al., 2021; Bamford et al., 2024). In particular, CNTN4 is considered to be the most involved in the development of autism among all contactins (Zuko et al., 2013). *Cntn4*-deficient mice show impaired brain morphology and autism-like behavior (Oguro-Ando et al., 2021; Wang et al., 2025). In human, deletions of this gene and nearby loci lead to the development of 3p deletion syndrome, which is characterized by multiple disorders, including intellectual disability (reviewed by Fernandez et al., 2008). Furthermore, case–control studies have shown that the *CNTN4* gene is associated with Alzheimer’s disease (Blacker et al., 2003) and schizophrenia (Fromer et al., 2016; Ma et al., 2018).

### 2.5 CNTN5

CNTN5 (NB-2), its key structural and expression features, was first described by Ogawa et al. (1996). This protein interacts with PTPRG, contactin-associated protein 4 (Caspr4), and amyloid precursor-like protein 1 (APLP1) as coreceptors (Shimoda et al., 2012; Mercati et al., 2013; Peng et al., 2017) and plays a role in the early neurodevelopment of both the brain and spinal cord. In the spinal cord, the CNTN5/Caspr4 complex interacts with neuronal cell adhesion molecule (NrCAM) and a close homolog of L1 (CHL1), and this interaction is associated with the accumulation of specialized GABApre bouton synapses (Ashrafi et al., 2014). In the mouse retina, the Cntn5/Caspr4 complex acts to anchor ON branch dendrites in ON–OFF direction-selective retinal ganglion cells (Peng et al., 2017). In addition, CNTN5 promotes the development of glutamatergic neurons in the auditory brainstem (Toyoshima et al., 2009). In accordance, *Cntn5*-deficient mice exhibit aberrant responses to acoustic stimuli (Li et al., 2003) and reduced number of fibers and synapses in the auditory system (Shimoda and Watanabe, 2009). In rodent auditory system Cntn5 expression is low in prenatal development, reached a maximum level in postnatal period, and declined in adulthood Ogawa et al., 2001; Toyoshima et al., 2009). In contrast, Kleijer et al. (2015) showed that Cntn5 starts to express actively at E15.5 and its levels persist into adulthood in the thalamus, caudate putamen and cerebral cortex. In adult human brain, CNTN5 is highly expressed in the amygdala and occipital lobe and low expressed in the corpus callosum, caudate nucleus, and spinal cord (Kamei et al., 2000). The role of variants in the *CNTN5* gene in the development of ASD has been shown in several studies (reviewed by Zuko et al., 2013). In addition to case reports (Van Daalen et al., 2011), most large-cohort studies (Poot, 2014; Mercati et al., 2016; Schmilovich et al., 2022), with one exception (Hadi et al., 2025), found the *CNTN5* variants more frequent in ASD patients than in healthy individuals.

Based on the above studies, it can be concluded that, despite the similarity of structure and coreceptors, members of the CNTN family have a specific role in neurodevelopment. CNTN1 and CNTN2 play the most important roles in the formation of neural networks and myelination in the embryonic period (Çolako Glu et al., 2014; Mohebiany et al., 2014). In adulthood, their expression levels decrease, and they are found only in certain areas of the brain, where they continue to perform the same functions at the local level. The role of CNTN3 remains open. Perhaps its functions and expression patterns differ significantly between humans and model animals (Yoshihara et al., 1994). The expression of Cntn4 has been detected in the prenatal period in rodents at very low levels, and this protein directs the development of neural networks in the early postnatal period, after which its expression decreases and remains constant throughout adulthood (Yoshihara et al., 1995). A number of studies showed that Cntn5 expression is also most active in the postnatal period, but the data is contradictory. Expression of contactins 1–5 has been studied primarily in adults, therefore the dynamics of expression during neurodevelopment are unclear. The localization of expression of contactins 1–5 in humans also does not always coincide with animal models. Variants and knockouts in the genes are associated with the development of various pathologies. The most clearly demonstrated association of CNTN1 (Shimoda and Watanabe, 2009) and CNTN2 (Ma et al., 2008) proteins of this family with myelination disorders and association of CNTN4 and CNTN5 (Zuko et al., 2013) proteins with the development of ASD. Several studies show a possible association of CNTN1 with myopathy (Compton et al., 2008), CNTN3 with tuberous sclerosis (Korotkov et al., 2022), CNTN2 with Alzheimer’s disease (Singh et al., 2015), and CNTN5 with intellectual disability (Zuko et al., 2013).

*CNTN6*, along with *CNTN5*, was first described in 1996 in the above work by Ogawa et al. (1996), later than most members of the family. It has attracted close attention due to its possible role in the direction of neurite growth and myelination and appears to have a more subtle effect on these processes in comparison with contactins 1–3. The next sections describe its properties and probable functions in detail.

## 3 CNTN6: structure, ligands, and cellular responses

*CNTN6* (*NB-3*) is evolutionarily closest to *CNTN3* and *CNTN4* (Ogawa et al., 1996). This gene is located on the short arm of chromosome 3 between the *CHL1* and *CNTN4* genes (Kamei et al., 1998). In rats, the *Cntn6* gene has 60% similarity to the *Cntn1* gene in nucleotide sequence and 42, 44, 58, 61 and 51% similarity to contactins 1, 2, 3, 4 and 5, respectively, in amino acid sequence (Ogawa et al., 1996). *CNTN6* is highly conserved and coincides in rats and humans by 90% (Kamei et al., 1998).

**Figure 1:**
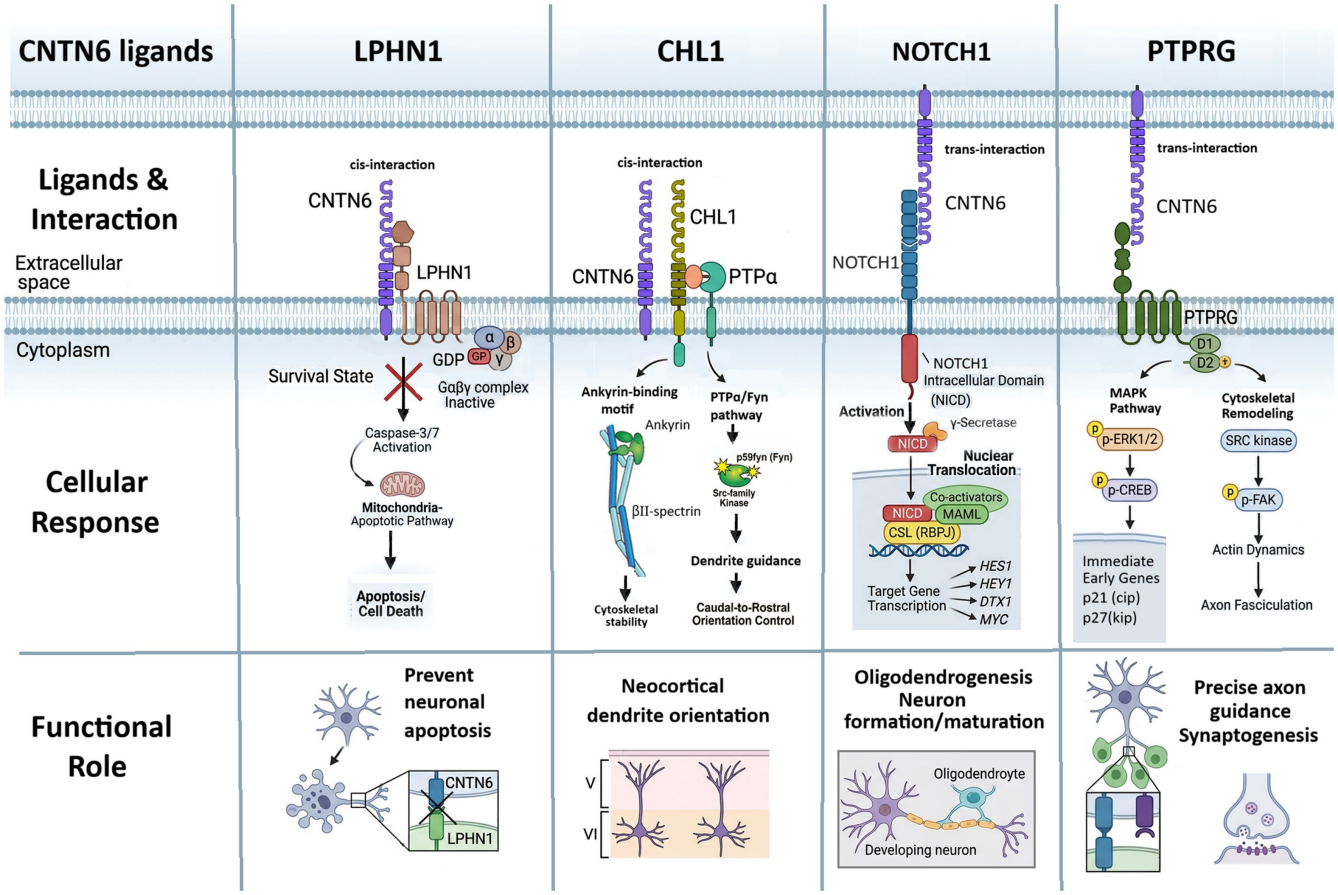
Schematic representation of known interactions of CNTN6 with other proteins, including LPHN1, CHL1, NOTCH1, and PTPRG. The CNTN6-LPHN1 cis-complex (within the same cell) is “silent” because it prevents LPHN1-mediated neuronal apoptosis. The CNTN6-CHL1 cis-complex includes PTPRɑ (Protein Tyrosine Phosphatase Receptor Type ɑ) and is critical for orienting the growth of apical dendrites. In the CNTN6-NOTCH1 interaction (cis- or trans-complexes), CNTN6 acts as a ligand for the NOTCH1 receptor; this interaction triggers the nuclear translocation of the Notch intracellular domain (NICD), driving gene expression for oligodendrocyte and neuron differentiation and maturation from neuronal progenitors. The CNTN6-PTPRG (cis- or trans-complexes) acts as a regulatory “switch” that translates extracellular cell-surface recognition into intracellular signaling by several downstream pathways. This interaction provides topographically precise neurite elongation and correct orientation of apical dendrites during neurodevelopment, and synaptic plasticity in the adult brain.

Like other contactins, CNTN6 consists of six N-terminal immunoglobulin (Ig) domains and four fibronectin type III (FNIII) domains (Zuko et al., 2016b), which are embedded to the outer side of the cell membrane with a glycosylphosphatidylinositol (GPI) anchor at the C-terminus (Ogawa et al., 1996). Due to this anchor, CNTN6 is able to form both cis- (same cell) and trans- (another cell) complexes. To date, several functionally distinct ligands and binding sites for CNTN6 have been identified. The molecular mechanisms of interactions and downstream signaling cascades of CNTN6 are schematically presented in Figure 1.

Through N-terminal binding center, CNTN6 interacts with CHL1 and creates a cis-complex, which interacts with alpha-tyrosine phosphatase (PtPɑ) and regulates apical orientation and dendritic growth in the developing caudal cortex (Huang et al., 2011; Ye et al., 2008). CNTN6 and CHL1 can shape a co-receptor complex to bind to a PTPα, adding flexibility and stability to the nervous system (Hu et al., 2003; Ye et al., 2008). The functionality of CNTN6 and CHL1 is not limited to this: individually, they independently bind to ligands and regulate dendrite development and orientation, making the process more stable (Huang et al., 2011). For example, CHL1 can bind to the βII to effect neurogenesis (Tian et al., 2012) and Semaphorin 3B and Neuropilin-2 and PlexinA4 to regulate dendritic spine density in the developing cerebral cortex (Mohan et al., 2019). In addition, CHL1 binds to the hedgehog receptor patched-1 to promote the survival of cerebellar granule cells during postnatal development (Katic et al., 2017). CHL1 is not a unique ligand of CNTN6, as it also shapes complexes with CNTN5 and NrCAM to form specific synapses (Nikolaienko et al., 2016).

Latrophilin-1 (Lphn1/ADGRL1) also forms a cis-complex with CNTN6 on the same cell membrane though GPI anchor. This interaction is “silencing” since CNTN6 acts as an endogenous ligand to prevent Lphn1-mediated apoptosis (Zuko et al., 2016a; Zuko et al., 2016b).

Additionally, CNTN6, as well as CNTN4 and CNTN5, binds to PTPRG through a conserved interaction surface, primarily involving Ig2-Ig3 domains (Nikolaienko et al., 2016). Despite similarity of conserved structure and a common PTPRG-binding site, CNTN3, 4, and 6 play distinct roles in neural network assembly (Zuko et al., 2013). CNTN4 primarily directs axon guidance and precise topographic mapping, particularly in the olfactory system (Kaneko-Goto et al., 2008) and accessory optic system (Osterhout et al., 2015). In contrast, CNTN6 specifically modulates excitatory VGLUT1/2-positive synaptogenesis at glutamatergic terminals in the cerebellum and hippocampus during postnatal development (Sakurai et al., 2009, 2010). Although CNTN3 has similar expression patterns in the cerebral cortex and hippocampus (Lein et al., 2007), it is not specialized for glutamatergic terminals like CNTN6. Thus, CNTN3/4 focus on projection specificity, while CNTN6 uniquely directs excitatory circuit refinement (Zuko et al., 2013). Due to the flexible Ig2 and Ig3 domains, CNTN6 can form both cis (on the same cell membrane) and trans (across different cells) complexes with PTPRG (Nikolaienko et al., 2016). Since PTPRG is transmembrane protein, it transmits signals into the cell initiating a phosphatase-depending signaling cascade to provide a precise axon guidance (Nikolaienko et al., 2016; Mercati et al., 2013).

There is growing evidence that axonal spatiotemporal signals modulate oligodendrocyte maturation and myelin formation. What role does CNTN6 play in this process? Cui et al. (2004) found that CNTN6 acts as a ligand for NOTCH1 involvement in oligodendrocyte generation in a pathway similar to that for CNTN1 (Hu et al., 2003, 2006). It has been shown that contactins 3–6 can embody complexes with PTPRG and affect Notch1 activity (Nikolaienko et al., 2016).

Notch receptors are evolutionary conserved transmembrane receptors. They transmit extracellular signals across the cell membrane and trigger signaling cascades that regulate gene expression. Notch consists of three domains: extracellular domain rich in epidermal growth factor repeats, single transmembrane domain, and intracellular domain NICD (Intra-Cellular Domain Notch) (Arias et al., 2002). CNTN6 molecules are localized on the axon membrane of the neuron, and Notch1 molecules are localized on the membrane of the neuronal progenitor cell. The extracellular domain of CNTN6 binds to and activates Notch1, and their interaction results in the cleavage of the Notch1 extracellular domain. The remaining intramembrane C-terminal fragment is processed by the *γ*-secretase complex. As a result of these events, the intracellular domain of NICD is eliminated at the S3 site via regulated intramembrane proteolysis (RIP) (Cui et al., 2004). In the cytoplasm of the future glial cell, NICD binds the ankyrin repeats of its intracellular domain to the N-terminus of the Deltex1 protein. The NICD-Deltex1 complex then translocates to the nucleus, where it modulates the expression of various target genes, such as *Hes* (Arias et al., 2002), and directly or indirectly mediates the expression of myelin-associated proteins. In oligodendrocyte precursors, CNTN6 increases the amount of myelin-associated glycoprotein (MAG) transcripts (Cui et al., 2004). MAG-mediated signaling from axons to oligodendrocytes is required for efficient myelination and maintenance of healthy mature oligodendroglia (Quarles, 2007). The important role of CNTN6 in the myelination process is also indicated by the timing of its expression in early postnatal development, which coincides with the timing of the main oligodendrocytogenesis (Kuhn et al., 2019).

## 4 CNTN6: spatial and temporal expression patterns in the normal brain

The majority of studies indicate that CNTN6 is expressed only in the central nervous system (Shimoda and Watanabe, 2009; Zuko et al., 2016b; Chatterjee et al., 2019). However, there are few studies of its expression in other organs. There is evidence of CNTN6 expression in the thyroid gland in mice and human (Zhang et al., 2024). In addition, Hu et al. (2015) noted that variants in the *CNTN6* gene often cause morphological abnormalities unrelated to the central nervous system.

The localization and timeline of Cntn6 expression in the mouse brain were studied in most detail (Kamei et al., 1998; Osterfield et al., 2008; Sakurai et al., 2009; Huang et al., 2012; Mu et al., 2018) (Table 1). Lee et al. (2000) showed that the level of *Cntn6* mRNA expression varies depending on the stage of individual development of mice. During embryogenesis, the level of *Cntn6* mRNA expression in the brain is low but begins to increase after birth and reaches a maximum at P7, which corresponds to the time of most active synaptogenesis. After P7 the expression decreases by approximately 80% of the maximum and remains stable throughout adulthood. The levels of *Cntn6* mRNA also vary across brain structures (Lee et al., 2000). The highest *Cntn6* mRNA level was found in the mouse hippocampus, mainly in CA1, which is associated with the active establishing new connections in this area at P7-P8 (Cossart and Khazipov, 2022). In the dentate gyrus and CA3, *Cntn6* mRNA levels increased by P14, although did not reach those in CA1 (Zuko et al., 2016b). In the visual cortex, Cntn6 was detected in mice at P14, but became negligible by adulthood. In the thalamic anterodorsal and anterovental nuclei, a similar level of Cntn6 was detected both at P14 and in adulthood (Zuko et al., 2016b). In the mouse cerebellum, the dynamics are strikingly different: the levels of *Cntn6* mRNA gradually increased from birth to 3 months, after which it remained stably high (9 times higher than at birth) throughout life (Lee et al., 2000).

We found only one study of CNTN6 expression in humans: the results of *in situ* hybridization on postmortem samples of adult brains showed high levels of expression in the cerebellum, thalamus, and subthalamic nucleus, and low levels in the corpus callosum, caudate nucleus, and spinal cord (Kamei et al., 1998). However, these results should be interpreted with caution, because the authors did not report important information about the study including the criteria and methods for sample collection, as well as sex, age, and medical history of the patients from whom samples were collected. We did not find any data on the localization and dynamics of CNTN6 expression in humans during the normal pre- and early postnatal periods in the available literature. In addition, changes in CNTN6 expression during aging were not investigated in either animals or humans.

Therefore, CNTN6 participation in the formation and maintenance of the neural network was shown for two directions: the guidance and branching of neurites and oligodendrocytogenesis. In early postnatal neurogenesis, CNTN6 is most important in the hippocampus and visual cortex, whereas in the cerebellum and cortex, it is actively expressed throughout life.

## 5 Neuropathology shown on animal models of CNTN6 deficiency

### 5.1 *Cntn6*-knockout mice

Animal models, primarily genetically modified mice with *Cntn6*^−/−^ genotype, are the main tool for investigating the dysfunctions of CNTN6 (Takeda et al., 2003; Sakurai et al., 2009; Huang et al., 2012; Korablev et al., 2017; Pristyazhnyuk et al., 2019; Zhang et al., 2024).

*Cntn6*-knockout mice are viable, fertile, have normal body and brain weights, and show no obvious differences from their WT littermates in either appearance or behavior (Takeda et al., 2003). In contrast to this study, Zhang et al. (2024) revealed a smaller body size and slower weight gain in *Cntn6*-knockout mice in comparison with WT mice. In addition, this study found hypothyroidism due to disrupted Notch1 signaling in *Cntn6*-knockout mice (Zhang et al., 2024). Despite the lack of visible behavioral changes, more subtle behavioral and physiological differences between these mice and their (WT) counterparts were identified in specific tests by a number of studies (Takeda et al., 2003; Mu et al., 2018; Zhang et al., 2024).

### 5.2 Impaired motor coordination

Takeda et al. (2003), who first created *Cntn6*-knockout mice, found impaired motor function in these mice, in particular, slower learning in the rotarod test, the dysfunction of equilibrium and vestibular senses. Differences compared to (WT) mice were found for motor coordination, but not for muscle strength. Sex differences in motor coordination were not examined in this study, as behavioral tests were performed only on males.

Based on Nissle histological staining and immunochemical staining with neurofilaments, Takeda et al. (2003) reported no obvious abnormality in the brain architecture of the *Cntn6*-deficient mice. However, several later studies have shown abnormal cerebellar development in these mice (Sakurai et al., 2009, 2010), which may explain the motor coordination impairments. Reduced synaptic terminal density of parallel fibers between parallel fibers and Purkinje cells was found in the developing cerebellum that caused an increase in caspase-dependent cell death (Sakurai et al., 2009). Using cultured neurons from *Cntn6*-deficient mice, Zuko et al. (2016a) also found increased neuronal apoptosis and reduction in neurite outgrowth depends on GPCR latrophilin-1 (Lphn1, a.k.a. CIRL1/CL, ADGRL1), which can be prevented by forming a heteromeric cis-complex of Cntn6 and Lphn1.

In addition to cerebellar abnormalities, *Cntn6*-knockout mice showed delayed corticospinal tract development, which may be associated with impaired motor coordination (Huang et al., 2012). The authors reported delays in projections from the motor cortex and terminal arborization of corticospinal axons at embryonic and early postnatal stages, while these processes were able to complete and corticospinal projections were not different from WT mice at P21.

### 5.3 Sex-dependent slower spatial learning

The study by Mu et al. (2018) has examined spatial learning and memory abilities of *Cntn6*-knockout mice in the Morris water maze taking into account sex of animals. Only male *Cntn6*^−/−^ mice showed slower learning than (WT) males, and only during the acquisition period of Morris water maze task but not in reversal spatial learning. *Cntn6*-knockout and heterozygous females showed no differences from (WT) females in the first phase of learning and even shorter platform reaching times during reversal learning. The authors interpret better performance of mutant females in the reversal task as a form of cognitive flexibility.

Since learning in the Morris water maze is hippocampus-dependent, it is important to see into the studies examined impairments in this brain structure. It is known that Cntn6 is expressed in the hippocampal regions including the CA1, DG and hilus (Lee et al., 2000; Zuko et al., 2016b). Zuko et al. (2016b) described abnormalities in the hippocampus of *Cntn6*^−/−^ mice in detail. The authors reported that the delineation and outgrowth of mossy fibers remained largely unchanged. The only abnormality that was found in the hippocampus was an enlargement of the suprapyramidal bundle of the hilus, as opposed to the infrapyramidal bundle. More abnormalities were found in the cortex: the number of projection neurons decreased in layers II-IV and increased in layer VI in the visual cortex, the number of parvalbumin+ GABAergic interneurons decreased, while the number of NPY + GABAergic interneurons remained unchanged. Unfortunately, such an important parameter as the sex of the mice, especially in the context of sex-dependent behavioral changes in Cntn6 deficiency, was not specified in this study.

### 5.4 Impaired reproductive behavior and olfactory function in *Cntn6-*deficient male mice

The effect of Cntn6 deficiency on male mice recently was demonstrated by Zhang et al. (2024). The reproductive behavior of *Cntn6*-deficient males was investigated in the urine sniffing and mate preference tests. *Cntn6*^−/−^ adult male mice showed less interest and reduced mating attempts toward estrous female mice in comparison with (WT) males. Since the accessory olfactory system is responsible for the detection of pheromones emitted by animals, Zhang et al. (2024) investigated the effect of Cntn6 deficiency on the microstructure and neuronal activation of the accessory olfactory bulb. They observed no difference between *Cntn6*^−/−^ and *Cntn6*^+/+^ mice in microstructure including vomeronasal sensory neurons and their axonal projections. However, *Cntn6*^−/−^ male mice showed an increased number of activated granule cells, detected by c-Fos, in the accessory olfactory bulb and decreased neural activity in the medial amygdala and medial preoptic area, which receive projections from the olfactory bulb, after female exposure.

### 5.5 Excitatory vs. inhibitory effect of CNTN6 deficiency

Although there are few studies investigating the role of CNTN6 in modulating neurotransmitter systems, available data point to its involvement in excitatory-inhibitory balance. Animal studies have shown that Cntn6 impacts both GABAergic and glutamatergic neurotransmission in the brain, but its effects on these two systems differ. Cntn6 has been shown to be expressed at presynaptic terminals of glutamatergic synapses in the hippocampus, where it interacts with vesicular glutamate transporter 1 (VGLUT1) and 2 (VGLUT2) that regulate glutamate uptake into presynaptic vesicles (Sakurai et al., 2009, 2010). Cntn6 deficiency reduces VGLUT1 and VGLUT2 expression in the hippocampus, suggesting that Cntn6 is involved in the formation and maintenance of glutamatergic synapses. The effect of Cntn6 on glutamatergic synapses was found to be selective, as *Cntn6*-knockout mice showed a decrease in the density of glutamate markers (VGLUT1, VGLUT2), but not the GABAergic marker (VGAT) (Sakurai et al., 2010). In contrast, Zuko et al. (2016b) found that Cntn6 deficiency is associated with a decrease in the number of GABAergic interneurons in the visual cortex and a decrease in GABA synapse density.

### 5.6 Brachycephaly of Persian cat is associated with CNTN6 deficiency

Selectively bred domestic animal breeds, although not animal models *per se*, can provide additional clues about the relationship between genes and morphological and behavioral traits. Bertolini et al. (2016) investigated genotypic features of the breed of Persian cat that distinguishes by specific phenotype including brachycephaly, large round eyes, small ears, long coat, and roundness of the body. The region of *Chl1* and *Cntn6* genes, located on chromosome A2 in cat, was found to have the highest homozygosity and the greatest divergence from the non-Persian breed among all examined regions. The authors suggest *Chl1* and *Cntn6* as candidate genes for the face morphology determination and could influence the behavior in Persian cat.

## 6 Pathologies associated with CNTN6 in human

Although studies on the role of CNTN6 in the pathogenesis of human diseases are still limited, apart from case reports (Li et al., 2016; Juan-Perez et al., 2018; Tassano et al., 2018; Thammachote et al., 2020; Pol-Fuster et al., 2021), more extensive studies using several different approaches can be identified.

The first approach (Table 2) is to estimate the *CNTN6* copy number variation (CNV) and/or single nucleotide variants (SNV) in a large population of patients with neuropsychiatric disorders and healthy individuals, often using public databases. The result of these studies is usually not only an assessment of the frequency of *CNTN6* mutations in these populations, but also a description of specific neuropsychiatric disorders in patients with *CNTN6* variants and in their family members. The second type, classical case/control studies, includes investigation of CNVs of the *CNTN6* gene in homogeneous cohorts of patients with ASD, ID, TS, and bipolar disorder (BD) (Table 3). A more sophisticated variant is to use Gene Ontology enrichment analysis (GOEA), where the association with a disease is examined not for a single gene, but for a functionally unified gene set. In particular, the *CNTN6* gene is one of 229 genes in the GO:0098742 category (cell–cell adhesion via plasma-membrane adhesion molecules) (Givogri et al., 2002; Kushima et al., 2018). The interpretation of such studies is complicated by the presence of comorbidities (for example, ASD with ID). A number of studies combine both of the above approaches (Repnikova et al., 2020). In addition, several studies are direct to understanding the pathophysiological basis associated with *CNTN6* mutation in humans, including psychometric measurements and analysis of patient-derived induced pluripotent cell (iPS) cultures.

**Table 2:** Prevalence of *CNTN6* variants in healthy individuals and patients with mental and neurodevelopmental disorders.

References	Cohort	Total sample, *N*	Variants, *N* (frequency)	Deletions/duplications	Diseases (*N* cases)	MD/ND, *N* (frequency)
Murdoch et al. (2015)	Control	942	16** (1.69%)	n.r.	–	–
Mercati et al. (2016)	Control	8,936	13** (0.15%)	1/12	–	–
Huang et al. (2017)	Control	4,100	2 (0.05%)	0/2	–	–
Kushima et al. (2018)	Control	2095	1 (0.05%)	1/0	–	–
Zarrei et al. (2023)	Participants* without MD/ND or high traits associated with ADHD, OCD, anxiety, and ASD	4,638	17 (0.37%)	3/14	Asthma (1) Hearing problems (2) Diabetes (1)	0 (0)
Zarrei et al. (2023)	Participants* with high traits associated with ADHD, OCD, anxiety, and ASD	1948	11 (0.56%)	2/9	ASD (2) ADHD (5) OCD (3) Anxiety (3)	8 (73%)
Zarrei et al. (2023)	Participants* with MD/ND	1,232	10 (0.81%)	2/8	ASD (2) ADHD (4) OCD (3) Tics (1) Anxiety (2) Learning problems (3)	10 (100%)
Hu et al. (2015)	Patients with multiple congenital anomalies, heart defect, short stature, DD, ID, ASD, seizures, or other unexplained anomalies	3,724	14 (0.38%)	7/7	ASD (3) DD (10) ADHD (4) Seizures (7) Cranial abnormalities (4) Digital anomalies (3) Eye problems (2)	13 (93%)
Repnikova et al. (2020)	Patients* with abnormal development	20,226	19 (0.09%)	6/13	ASD (7) DD (9) ADHD (6) Hypotonia (3) Seizures (3) Cardiac abnormalities (5) Low weight (5) Cranial abnormalities (2) Digital anomalies (2)	15 (79%)

*, Children, **, rare variants including missense, nonsense, splice site, start or stop codon ones, n.r., not reported; MD, mental disorder; ND, neurodevelopmental disorder.

**Table 3:** Prevalence of *CNTN6* variants in patients with ASD, ID, ADHD, TS, BD, anorexia nervosa, and anxiety.

Disease	Total sample, *N*	*CNTN6* variants, *N*, (del/dup)	Isolated variants, *N*, (del/dup)	Frequency, % (del/dup)	Sex, m/f	Test vs. controls, statistics	References
ASD	210^a^	2 (1/1)	1 (0/1)	0.95 (0.48/0.48)	2/0	NA	Van Daalen et al. (2011)
	144^a^	2 (2/0)	2 (2/0)	1.39 (1.39/0)	2/0	GOEA, fold = 8.32, FDR = 0.009^e^	Costa et al. (2022)
	194	3^d^ (0/3^d^)	NA	1.55 (0/1.55)	n.r.	NA	Nava et al. (2014)
	1,030	17^b^ (n.r.)	n.r.	0.017 (n.r.)	n.r.	Fisher’s, 0.4	Murdoch et al. (2015)
	1,534	8^b^ (6/2/18^c^)	2 (2/0)	0.52 (0.39/0.13)	8/0	Fisher’s, 0.00006/n.s./0.0005	Mercati et al. (2016)
	94^a^	0 (0/0/4^c^)	0 (0/0/4^c^)	0 (0/0)	n.r.	Hotelling’s T2, n.s.	Repnikova et al. (2020)
	145^a^	2 (1/1)	2 (1/1)	1.38 (0.69/0.69)	1/1	Chi-square, n.s.	Zarrei et al. (2023)
	1,108	5 (4/1)	4 (4/0)	0.45 (0.36/0.09)	3/2	GOEA, OR = 1.6, q < 0.001^e^	Kushima et al. (2018)
ID	79	4^e^ (3^e^/1)	4^e^ (3^e^/1)	3.8 (2.53/1.2)	2/2	NA	Kashevarova et al. (2014)
	200	0 (0/0)	0 (0/0)	0 (0/0)	–	NA	Lopatkina et al. (2016)
TS	2,435	13 (1/12)	10 (1/9)	0.53 (0.04/0.49)	12/1	Fisher’s, 0.00025	Huang et al. (2017)
	1,086	8 (0/8)	6 (0/2)	0.74 (0/0.74)	n.r.	Fisher’s, n.s.	McGrath et al. (2014)
SCZ	2,458	1 (1/0)	1 (1/0)	0.04 (0.04/0)	1/0	GOEA, n.s.^e^	Kushima et al. (2018)
Anorexia nervosa	1,033	3 (1/2)	1 (0/1)	0.29 (0.1/0.19)	n.r.^f^	Fisher’s, n.s.	Wang et al. (2010)
BD	936	2 (2/0)	1 (1/0)	0.21 (0.21/0)	n.r.	NA	Noor et al. (2014)
ADHD	474^a^	4 (1/3)	4 (1/3)	0.84 (0.21/0.63)	3/1	Chi-square, n.s.	Zarrei et al. (2023)
Anxiety	320^a^	3 (1/2)	3 (1/2)	0.93 (0.31/0.63)	2/1	Chi-square, n.s.	Zarrei et al. (2023)

^a^Children, ^b^number of cases with rare variants including missense, nonsense, splice site, start or stop codon ones; ^c^number of cases with rare SNVs; ^d^between CNTN4 and CNTN6; ^e^two siblings, male and female; ^f^1,009 of 1,033 patients were women; ^е^for gene set, n.r., not reported; NA, data not available.

### 6.1 *CNTN6* variants in the cohorts of neuropsychiatric patients and healthy individuals

Most studies that examined their own samples or public databases found *CNTN6* variants in healthy individuals (Table 2). The frequency of *CNTN6* gene variants in healthy individuals identified in different studies varies hundreds of times (e.g., 3.2% in the study by Murdoch et al. (2015) and 0.05% in the study by Huang et al. (2017). These discrepancies are likely due to differences in sample sizes, types of variants examined (CNVs, SNVs, rare variants, microdeletions, etc.), and mutation detection methods (microarrays, FISH, qPCR, etc.). At least, it can be stated that most studies based on large samples have shown that the frequency of *CNTN6* variants in healthy individuals, although small but not zero. Animal models also suggest that isolated *Cntn6* variants do not result in visible behavioral changes; these changes can only be revealed through specific behavioral experiments (Takeda et al., 2003).

The studies, which used large patient cohorts instead healthy individuals (Table 2), have shown that *CNTN6* variants are associated with multiple neurodevelopmental disorders including ASD, developmental delay (DD), attention-deficit/hyperactivity disorder (ADHD), obsessive-compulsive disorder (OCD), seizures, tics, hypotonia, cranial and digital abnormalities, hearing and vision problems (Hu et al., 2015; Repnikova et al., 2020; Zarrei et al., 2023). The study by Hu et al. (2015) revealed 14 CNVs of *CNTN6* gene (7 deletions and 7 duplications) among 3,724 patients. Thirteen of these 14 patients (93%) with mutations had neurodevelopmental disorders. Repnikova et al. (2020) found 19 mutations (6 deletions, 13 duplications) in the *CNTN6* gene among 20,226 patients of children’s hospitals and 15 of these 19 patients (79%) had neurodevelopmental disorders. Only one of the above CNV studies compared the frequency of *CNTN6* variants in patients with controls in public databases and showed no significant differences between the samples (Repnikova et al., 2020). Both above studies found neuropsychiatric and neurodevelopmental disorders in family members of most, but not all, patients. *CNTN6* variants were mostly inherited from parents, but in some cases their occurrence *de novo* has been shown (Van Daalen et al., 2011; Murdoch et al., 2015; Mercati et al., 2016). Unfortunately, the frequency of *CNTN6* variants among patients with a specific disease (ASD, cranial abnormalities, etc.) has not been assessed in these large samples with known medical histories excluding the recent CNV study by Zarrei et al. (2023). This study is an excellent example of a comprehensive study of a large sample, including both healthy children and children with mental and neuropsychiatric disorders. All 7,100 children, in addition to self-reporting their diagnosis, completed a questionnaire, cognitive task, and stop-signal task to assess traits associated with ADHD, OCD, anxiety and ASD. Interestingly, *CNTN6* variants were found in only 0.37% of participants without mental disorders or high traits associated with ADHD, OCD, anxiety and ASD, in 0.81% of participants with mental or neurodevelopmental diagnoses, while participants with high-trait scores occupied an intermediate position at 0.56% (Table 2).

The case–control studies described below have assessed the association of *CNTN6* variants with specific mental disorders (Table 3).

### 6.2 Associations of *CNTN6* gene with specific neuropsychiatric and neurodevelopmental disorders in case–control studies

The relationship between *CNTN6* variants and ASD has been the most extensively studied (Table 3). Most studies, including several case reports (Li et al., 2016; Tassano et al., 2018; García-Ortiz et al., 2020) and large-cohort studies (Van Daalen et al., 2011; Nava et al., 2014; Murdoch et al., 2015; Mercati et al., 2016; Kushima et al., 2018; Repnikova et al., 2020; Costa et al., 2022), have shown that *CNTN6* variants induces the presence of the ASD phenotype, although not directly.

Van Daalen et al. (2011) found 2 *CNTN6* variants (1 deletion, 1 *de novo* duplication) in 210 children with ASD. Based on patient pedigree data, van Daalen showed that hemizygosity for the *CNTN6* gene alone may not be sufficient to develop ASD, and that loss of *CHL1* and part of the *CNTN6* gene may cause a weaker phenotypic effect than gain of part of this gene. The authors concluded that *CNTN6* mutations do not cause ASD *per se* but only contribute to the manifestation of the autistic phenotype. Nava et al. (2014) found an intergenic duplication Nava et al. (2014) found an intergenic duplication between *CNTN4* and *CNTN6* in three of 194 ASD patients. Costa et al. (2022) studied rare CNVs in the ASD cohort (144 patients) and revealed enrichment of the *CNTN6* gene along with 14 other significant ASD candidate genes using GOEA. Zarrei et al. (2023) identified *CNTN6* deletions (0.69%) and duplications (0.69%) as a significant susceptibility CNV for ASD. Repnikova et al. (2020) found only three rare SNVs in four of 94 patients with ASD and found no differences compared to controls. The authors concluded that mutations in the *CNTN6* gene are likely neutral variants or modifiers but do not cause disease.

Case–control studies on large cohorts of ASD patients (>1,000) have similar results. Kushima et al. (2018) found 5 *CNTN6* variants (4 deletions, 1 duplication) in 1108 ASD patients. Enrichment of the GO:0098742 gene set, which includes *CNTN6* gene, was found to be significant for ASD but not for SCZ. Mercati et al. (2016) in a large sample of 1,534 ASD patients and 8,936 healthy individuals found that *CNTN6* variants are significantly more common in ASD patients than in controls (approximately 1.7% vs. 0.15%). The authors also showed an association between rare *CNTN6* variants and auditory hypersensitivity and motor coordination impairments in ASD patients (Mercati et al., 2016). In contrast, Murdoch et al. (2015) did not find an increase in the frequency of rare *CNTN6* variants in ASD patients compared to controls. Mercati suggests the cause of these discrepancies: Murdoch et al. (2015) used the Simons Simplex Collection, which excludes first-degree relatives with ASD (Mercati et al., 2016). Therefore, most studies have confirmed the association of *CNTN6* variants with ASD.

Autism is often accompanied by intellectual disability (ID) (Kashevarova et al., 2014; Mercati et al., 2016; Tassano et al., 2018; Costa et al., 2022) and the incidence of these cases varies quite widely (e.g., 22% according to (Costa et al., 2022), 57% according to (Mercati et al., 2016)). Kashevarova et al. (2014) detected the microdeletion of the *CNTN6* gene in two siblings, and the microduplication in one patient out of 79 patients with ID. Further studies of this research group found no *CNTN6* variants in 200 patients with ID including 154 patients with idiopathic ID (Lopatkina et al., 2016) (Table 3). Based on the descriptions of 40 publicly available cases the authors estimated *CNTN6* variants in the patients with idiopathic ID as 0.4%. However, the potential association of *CNTN6* with ID remains preliminary and unconfirmed in large-cohort studies.

Although the number of studies is still few (McGrath et al., 2014; Huang et al., 2017) association of *CNTN6* with Tourette syndrome (TS) has been most clearly demonstrated. Moreover, studies on large cohorts of patients have shown that TS is associated specifically with *CNTN6* duplications. In a GWAS of rare CNVs in obsessive-compulsive disorder (OCD) and TS, McGrath et al. (2014) reported 8 exonic duplications, including 6 isolated *CNTN6* duplications, in 1086 patients with TS. Six of these 8 TS patients also had OCD. The frequency of duplications in these patients (0.72%) was higher than in controls (0.33%), but no significant differences were found. In contrast, Huang et al. (2017) in a CNV study found that duplications of the *CNTN6* gene significantly increased the risk of developing TS. Twelve exonic *CNTN6* duplications (0.49%) were detected in 2,434 patients with TS and only 2 duplications (0.05%) in 4,093 controls. Interestingly, the *CNTN6* duplications in TS cases were considerably larger than those in controls (641 vs. 143 kb); however, three of them also included the *CNTN4* gene. Although the study by Huang et al. (2017) was conducted on a large cohort of patients, it is the only one to show an association between TS and *CNTN6* duplications; further studies are needed to confirm this finding.

Several case reports (Juan-Perez et al., 2018; Thammachote et al., 2020; Toyama et al., 2022), large-cohort studies of patients with neuropsychiatric disorders (Hu et al., 2015), and one study of the large cohort of the patients with SCZ (Kushima et al., 2018) found the variants in *CNTN6* gene in SCZ patients. Juan-Perez et al. (2018) described *CNTN6* deletion in patient with SCZ, generalised epilepsy, and digital abnormalities. Thammachote et al. (2020) reported female patient with SCZ, poor insight and compliance, visual and auditory hallucination and deletion encompassing *CNTN4* and *CNTN6*. Toyama et al. (2022) examined 14 families with multiple cases of SCZ, and only in one family with a consanguineous marriage, a homozygous mutation in the *CNTN6* gene was found to be associated with SCZ. Pol-Fuster et al. (2021) described familial psychosis with a rare variant in the MACF1 gene combined with duplications in *CNTN6* and *CDH13* genes. In the study by Hu et al. (2015), 2 out of 14 patients with *CNTN6* mutations had family members with SCZ. In a comparative CNV study of *CNTN6* variants in patients with ASD and SCZ, Kushima et al. (2018) found only one *CNTN6* deletion in 2458 patients with SCZ (0.04%) and no significant difference with controls (0.05%). In GWAS, Zhao et al. (2013) examined 146 SNPs in the *CNTN6* gene for their association with schizophrenia. One significant SNP was identified, but no statistically significant association was found (Zhao et al., 2013). Although some case-reports found *CNTN6* variants in SCZ patients (Juan-Perez et al., 2018; Pol-Fuster et al., 2021; Thammachote et al., 2020; Toyama et al., 2022), two large-cohort studies rather indicate a weak association between SCZ and *CNTN6* mutations (Kushima et al., 2018; Zhao et al., 2013).

Data on the association of *CNTN6* with other neuropsychiatric disorders, in particular ADHD, anxiety, BD, and anorexia, are extremely limited. We attempted to collect this information from several GWAS studies not specifically focused on contactins (Table 3).

Zarrei et al. (2023) in a CNV study detected 4 isolated *CNTN6* variants (0.84%) in 474 patients with ADHD and 3 isolated *CNTN6* variants (0.93%) in 320 patients with anxiety. For both diseases, these frequencies were higher than in children without reported mental or neurodevelopmental disorders (0.37%), but the associations with disorder were not significant (Table 3). ADHD has been diagnosed in patients with *CNTN6* variants according to several other studies (Hu et al., 2015; Repnikova et al., 2020), although the frequency of mutations in ADHD patients has not been reported.

GWAS of SNP and CNVs in 936 patients with BD (Noor et al., 2014) detected 2 deletions (1 multigen and 1 single-gene) in the patients but no statistically significant association was found.

Wang et al. (2010) genotyped 1,033 patients with anorexia and found one case of deletion and 2 duplications of the *CNTN6* gene. The frequency of variants in the group of 3,733 controls was lower (0.03% vs. 0.29% in patients) but not significant.

Although congenital hypothyroidism is a metabolic disorder rather than a neuropsychiatric disorder, it results in growth retardation and neurological impairment. Zhang et al. (2024) identified three pathogenic variants of the *CNTN6* gene in two of 599 patients with congenital hypothyroidism.

### 6.3 Investigation of pathophysiological basis of *CNTN6* variants in human

Since the functional role of *CNTN6* gene in the pathogenesis of neuropsychiatric diseases is still unclear, studies aimed at identifying this role are extremely important. An excellent example is the study by Mercati et al. (2016), in which differences in questionnaire related to excessive sensibility to noise between carriers and non-carriers of *CNTN6* variants prompted to perform an objective audiometric study including otoscopic examination, tympanogram and a measurement of the stapedian ipsilateral reflexes. As a result, the authors revealed that probands carrying *CNTN6* variants were significantly more prone to suffer from hyperacusis, displaying more abnormal idiosyncratic-negative response to specific sensory stimuli than controls.

Another approach involves reprogramming technologies with an investigation of iPS cell cultures obtained from patients with *CNTN6* gene variant and a specific disease (Gridina et al., 2018, 2019, 2025; Shnaider et al., 2019). Gridina et al. (2019, 2025), and Shnaider et al. (2019) obtained a set of iPS cell lines derived from a patient carrier of the *CNTN6* gene duplication and from two healthy donors. These cell lines were differentiated into neurons and demonstrated characteristics of mature neurons both electrophysiologically and by the presence of key neuronal markers, specific to three germ layers (Gridina et al., 2019; Shnaider et al., 2019). Interestingly, the expression level of the inherited paternal duplicated allele was significantly reduced compared to both the expression level of the maternal (WT) allele and the maternal and paternal alleles in iPS cells from healthy donors (Gridina et al., 2018). The authors conclude that *CNTN6* expression is allele-specific and suggest a mechanism similar to genomic imprinting in the inheritance of *CNTN6* duplication. This hypothesis is supported by the fact that the healthy father of the donor of mutant iPS cells carries *CNTN6* duplication inherited from his mother. Therefore, maternal duplicated allele expresses CNTN6 in the healthy father. In further studies, the above-mentioned group of authors investigated the change in expression upon genome correction by deletion of the duplicate copy of *CNTN6* in the same iPS cell line (Gridina et al., 2025). However, even after the precise removal of one of the duplicated copies, *CNTN6* gene expression remained lower than in control cells and was comparable to the levels observed in the original cells before correction (Gridina et al., 2025). This indicates that epigenetic modifications and the transcriptional status of the corrected *CNTN6* gene copy are preserved even after the cells become pluripotent and undergo reprogramming.

## 7 Insights from animal models and human genetics

Based on the numerous genetic studies discussed above, it can be concluded that the *CNTN6* gene is involved in many neuropathologies. Its role in ASD and TS is the most well-documented. However, the molecular mechanisms of *CNTN6* aberrations have been studied primarily in animal models. Extrapolation of results from animal studies to humans is important for translational purposes. This question can be considered in terms of interspecies similarities in structure, ligand interactions, expression patterns, and phenotypic changes upon gene dysfunction.

CNTN6 is a highly conserved protein across species, including humans, mice, and rats. It maintains similar structural domains (six immunoglobulin-like domains, four fibronectin type III domains, and a GPI-anchor) across species. Differences in structure between species primarily manifest as subtle variations in sequence, which affect protein–protein interaction interfaces and expression patterns during brain development, rather than completely different overall, molecular, or structural architecture (Zuko et al., 2011; Mercati et al., 2013). The protein sequence alignment between human and mouse is approximately 89.1% (Research Institute of Tsinghua, Pearl River Delta, 2023) that indicates similarity of the binding interfaces for its major receptors. However, subtle differences in these sequences can influence the stability and affinity of the resulting protein complexes. The interaction between CNTN6 and PTPRG through the Ig2-Ig3 domains is strictly identical in both human and mouse (Nikolaienko et al., 2016). The core of binding sites of the interactions of CNTN6 with CHL1, Notch receptor, and LPHN1 are highly conserved but not identical. The expression timing and local concentration of Notch1 differ between human and mouse developing brains (Yakovleva et al., 2025). In humans, NOTCH1 is more critical for early neurodevelopment and progenitor proliferation than in mice (Yakovleva et al., 2025).

Based on the similarity of contactin interactions with ligands in animal models and humans, it can be assumed that the disruptions of these interactions shown in knockout mice have similar functional consequences.

According to genetic studies on animals and humans, CNTN6 deficiency may cause ssignificant neurodevelopmental and physiological dysfunctions by disrupting its interaction with partner molecules PTPRG, Notch receptors, LPHN1, and CHL1. Deficiency in CNTN6 leads to blocked release and nuclear translocation of the NICD, impairing NOTCH1 transcriptional activity. Disruption of the CNTN6-Notch1 signaling pathway in the brain can result in impaired oligodendrocyte differentiation and myelination, impaired neurogenesis, stem cell depletion, and increased apoptotic cell death neural progenitors (Yakovleva et al., 2025). The loss of the CNTN6-PTPRG complex affects the organization of the axon in early central nervous system development (Zuko et al., 2013). Disruption of CHL1 and CNTN6 interaction results in misoriented dendrites in cortical pyramidal neurons (Mercati et al., 2016). The loss of CNTN6-LPHN1 interaction results in reduced neurite outgrowth and increased apoptosis (Zuko et al., 2016a). Given the specificity for glutamatergic (excitatory) synapses, CNTN6 deficit may result in reduced glutamatergic synapse density, while inhibitory (GABAergic) synapses remain unaltered. An imbalance in the glutamate/GABA ratio was shown to contribute to the pathophysiology of ASD (Hollestein et al., 2023) and ADHD (Edden et al., 2012).

However, despite the severity of these predicted dysfunctions, the *in vivo* physiology is more complex. Unlike *Cntn1* and *Cntn2*, *Cntn6*-knockout mice do not exhibit premature death (*Cntn1* knockouts, Table 1) or visible neurological impairment (seizures in *Cntn2* knockouts, Table 1). Moreover, the *CNTN6* variants are quite common in healthy individuals, without neurological or psychiatric consequences (Tables 2, 3). Therefore, potential compensatory mechanisms that mask the phenotype associated with the *CNTN6* mutations should be considered. Masking or compensation can occur both at the functional (replacement with other ligands at different levels of descending pathways) and at the genetic level.

In cases of *CNTN6* mutations, redundancy within the contactin family may partially compensate for its loss. At least, CNTN4 and CNTN5 have similar roles in axon guidance and neurite outgrowth (Chatterjee et al., 2018) and could prevent neurological consequences. CNTN6’s important synapse-forming function is also redundant: many other neuronal adhesion molecules (e.g., NCAM or other members of the L1 superfamily) could compensate for its absence (Duncan et al., 2021). By acting as a NOTCH1 ligand, CNTN6 is involved in oligodendrocyte formation and promotes NOTCH1 activation. In the absence of CNTN6, other members of the Notch ligand family, the Delta-like (DLL1, DLL3, DLL4) and Jagged (JAG1, JAG2) families, can assume this role (D'Souza et al., 2010). Although *in vitro* studies showed that *Cntn6*-deficient cell cultures caused severe abnormalities (Zuko et al., 2016a), *Cntn6*-knockout mice exhibited milder behavioral changes (Sakurai et al., 2009; Sakurai et al., 2010) and sex-dependent phenotype in behavioral studies (Zhang et al., 2024; Mu et al., 2018). This discrepancy may be explained by the presence of other adhesion molecules that form initial neural circuits during the period of low expression in the embryonic period, effectively compensating for the loss. The sex-specific negative effect of the *CNTN6* deletion on males observed in knockout models may be explained by the incomplete penetrance shown in human studies described below.

Beyond protein-level compensation, genetic and epigenetic factors can also modulate the clinical outcome. Interestingly, the phenomenon has been observed in humans where variants in *CNTN4* and *CNTN6* manifest as disease when they are inherited from fathers who are healthy carriers of the same variant, but not from mothers whose offspring-carriers are healthy as well (Kashevarova et al., 2014; Zhang et al., 2020). At the genetic level, potential mechanisms masking *CNTN6* variant effects may include sex-dependent incomplete penetrance and reduced expression of duplicated alleles, which have been confirmed by several studies.

Incomplete penetrance of chromosomal microdeletions and microduplications remains a critical unresolved issue, significantly impacting the assessment of CNV pathogenicity and genetic counseling, particularly in cases of inherited chromosomal variants. Beyond genomic imprinting and allele-specific gene expression, other epigenetic mechanisms that could influence the active or inactive chromatin state within the CNV region should be considered. These include skewed X-chromosome inactivation for maternally inherited CNVs located on the X chromosome, which provides evidence for a protective effect on maternal health but poses a disease risk for a son inheriting a single affected X chromosome from his mother (Tolmacheva et al., 2025). Furthermore, a reduced DNA methylation index and increased *IMMP2L* expression were observed in lymphocytes from healthy mothers compared to affected probands in families with maternally inherited 7q31.1 microdeletions associated with autism spectrum disorders (Vasilyev et al., 2021). The meiotic mismatch methylation (3 M) hypothesis (Pembrey et al., 2015) proposes another possible epigenetic mechanism. It is related to early meiosis I, where pairing of a chromosome carrying a microdeletion or microduplication with its wild-type homolog increases the chance of abnormal methylation due to chromosome looping from misaligned pairing. It is noteworthy that sex differences exist in the timing of epigenetic genome reprogramming and chromosome pairing during meiosis, which may explain parent-of-origin effects in gene expression influenced by CNVs (Skryabin et al., 2017). Finally, the “two-hit hypothesis,” which posits a pre-existing genetic background, should also be considered to explain the clinical manifestation and incomplete penetrance of genomic disorders (Girirajan and Eichler, 2010).

The studies on iPSC-derived neurons from a patient with a *CNTN6* duplication demonstrated that the duplication does not lead to a corresponding increase in expression (Gridina et al., 2018). On the contrary, the expression level of the duplicated allele was significantly reduced compared to the wild-type allele. Duplication in this family was inherited from a healthy father and grandmother and further studies revealed that *CNTN6* expression levels were comparable in neurons derived from the father’s iPSCs and those of healthy donors (Gridina et al., 2019a). Moreover, statistically significant differences were observed in the ratio of maternal and paternal *CNTN6* allele transcripts. In neurons derived from a healthy donor with a normal karyotype, expression of the maternal allele was slightly higher than that of the paternal allele (Gridina et al., 2018). These findings align with the hypothesis of a parent-of-origin effect on gene expression and may explain the incomplete penetrance of the microduplication when it is inherited from mother to son. While this inheritance pattern resembles genomic imprinting, there is currently no evidence for imprinting of the *CNTN6* gene. At the same time, evidence for parental bias in gene expression has been obtained for another member of the contactin family, CNTN5 (Santoni et al., 2017).

In summary, the high structural and functional conservation of CNTN6 between humans and mice supports the use of animal models to study its role in neurodevelopment. However, the relatively mild and sex-dependent phenotype in *Cntn6*-knockout mice and the existence of healthy human carriers indicate that CNTN6-related pathologies are strongly modulated by compensatory mechanisms. Parent-of-origin effect may determine the clinical outcome, highlighting the need to look beyond the primary genetic lesion when assessing pathogenicity.

## 8 General discussion and future directions

Since *CNTN6* was discovered later than other members of the contactins family, there are still few human genetic studies to draw conclusions about the involvement of the *CNTN6* gene in specific neuropsychiatric diseases, and small number of animal studies do not allow to understand pathological mechanisms associated with this gene. The fact that *CNTN6* variants (both deletions and duplications) occur in healthy individuals (Mercati et al., 2016; Huang et al., 2017; Kushima et al., 2018), and mice with *Cntn6* deletion have phenotype, visually indistinguishable from (WT) mice (Takeda et al., 2003), indicates that *CNTN6* is not a key gene responsible for any certain mental or neurodevelopment diseases. At the same time, many researchers consider *CNTN6* as a candidate risk gene, modifier or susceptibility gene for many neuropsychiatric disorders including ASD, ID, TS, and anorexia (Wang et al., 2010; Van Daalen et al., 2011; Kashevarova et al., 2014; Hu et al., 2015; Lopatkina et al., 2016; Mercati et al., 2016; Huang et al., 2017; Kushima et al., 2018; Repnikova et al., 2020; Costa et al., 2022).

Despite a number of studies including large cohorts of patients (Murdoch et al., 2015; Mercati et al., 2016; Kushima et al., 2018), the question of the relationship between *CNTN6* and ASD remains open. In most of these studies, the number of deletions in ASD patients exceeded the number of duplications, and single-gene deletions included only *CNTN6* were detected (Table 3). We have not found any studies of social behavior in mice with *Cntn6* deletion in the available literature, which could be directly related to the associations between this gene and ASD, except for impaired reproductive behavior in males, which the authors associate with olfactory disfunction (Zhang et al., 2023). Male *Cntn6*-deficient mice also showed slower spatial learning in the first phase of Morris maze task but not in reversal learning (Mu et al., 2018). Spatial navigation in ASD patients is generally intact but has some specific features: they demonstrate strengths within small-scale visuospatial or large-scale perceptual domains but show impairments on large-scale visuospatial domains (Smith, 2015). Of particular note is the abnormal response of ASD patients with *CNTN6* variants to auditory sensory stimuli, as observed by Mercati et al. (2016) in psychometric experiments. This psychophysiological feature could be studied in *Cntn6-*deficient mice, which may help clarify the role of this gene in ASD.

In contrast to ASD, TS is rather associated with duplications of the *CNTN6* gene (McGrath et al., 2014; Huang et al., 2017), most of which have also been isolated. TS is a hereditary neurodevelopmental disease that is characterized by chronic involuntary motor or vocal tics (Johnson et al., 2022). Interestingly, movement impairments, although of a different type, are observed in mice with a deletion, but not with a duplication of *Cntn6* (Takeda et al., 2003). To our knowledge, studies on a mouse line with a duplication of the *Cntn6* gene have not yet been conducted, although such mice were created using CRISPR/Cas9 technology (Korablev et al., 2017; Pristyazhnyuk et al., 2019). Mice with *Cntn6* duplication were viable, fertile, and showed no visible abnormalities. However, this mice strain has not been studied in behavioral experiments that could clarify the association between *CNTN6* duplication and neurological disorders including TS.

However, the studies (Gridina et al., 2018) on the iPS cell line from a patient with a duplication of *CNTN6* have shown that the duplication does not lead to an increase in the expression level. On the contrary, the expression level of the duplication allele was significantly reduced compared to the (WT) allele. The investigation of a mouse line with a duplication of the *CNTN6* gene and behavioral experiments on them could clarify the association of this gene with TS.

There are too few studies of the *CNTN6* gene to assess its contribution to SCZ, BD, ADHD, anxiety, and anorexia. It should be noted that most large-cohort genetic studies performed on children (Table 3) whereas the diagnosis of SCZ or BD is usually made in adolescence or adulthood (Baldessarini et al., 2012; Kendhari et al., 2016), thereby the association with these diseases may have remained out the focus of research. According to studies in animal models, *CNTN6* is highly expressed in adulthood (Lee et al., 2000), so a relationship between its alterations and these diseases is quite possible.

Given the potential involvement of *CNTN6* in various neuropsychiatric disorders and the fact that these disorders often co-occur, animal studies that could help understand the pathophysiological basis of *CNTN6* gene mutations are of great importance. Generally, CNTN6 has been shown to be involved in the guidance and branching of neurite outgrowth (Shimoda and Watanabe, 2009; Zuko et al., 2013, 2016b; Mohebiany et al., 2014), as well as in the regulation of oligodendrogenesis (Cui et al., 2004; Hu et al., 2006). Furthermore, CNTN6 expression levels in different brain regions vary throughout life and it is unclear which of the above functions and in which brain regions this glycoprotein has a greater influence. It is possible to assume different roles of this gene in neurodevelopment (guidance and neurite outgrowth) and maintenance of neural networks in adulthood (oligodendrogenesis and myelination). Creating and studying conditional knockout lines with gene inactivation during early postnatal development or adulthood could help clarify this question. Furthermore, using Cre-recombinase under the control of a region-specific promoter (for example, Emx1 for cortex/hippocampus (Alia et al., 2022), CaMKIIα for forebrain glutamatergic neurons (Gavériaux-Ruff and Kieffer, 2007)) would help clarify the *CNTN6* gene significance for specific brain regions. There are few morphological studies in *Cntn6*-deficient mice that have clearly demonstrated changes in axonal growth at different stages of development in different brain regions and no information on changes in myelination directly related to oligodendrogenesis. At the same time, abnormal myelination and connectivity have been found in many neurodevelopmental and psychiatric disorders. Quantitative MRI, including diffusion tensor imaging (DTI) and macromolecular proton fraction (MPF) mapping showed disrupted connectivity and demyelination in ASD, TS, and SCZ (Libero, 2014; Lee et al., 2006, 2007; Cavanna et al., 2010; Smirnova et al., 2021). Future quantitative MRI and fMRI studies in individuals with *CNTN6* variants and MR-histological studies, live-cell imaging, electrophysiology of brain slices and cell-cultures in animal models may clarify how the loss of this gene’s normal function in myelination and connectivity leads to neuropsychiatric disorders.

## 9 Conclusion

CNTN6 is the most recently discovered member of the contactins family that is expressed primarily in the CNS. This protein is expressed most intensely in the early postnatal period, but its expression remains high in adulthood in the cerebral cortex and cerebellum. The known functions of CNTN6 are related to neurite outgrowth guidance, oligodendrocytogenesis, and neural network formation and maintenance. *Cntn6*-deficient mice exhibit impaired motor coordination, sex-dependent slower spatial learning, and sex-dependent impaired reproductive behavior. Mutations in the *CNTN6* gene are a risk factor for various neurodevelopment and mental diseases including ASD, TS, ID, and SCZ. The associations of *CNTN6* with ASD and TS are most clearly demonstrated. Future studies may focus on social behavior and morphological changes in neural networks and myelination in animal models, as well as psychophysiological and quantitative MRI studies in patients with neuropsychiatric disorders and *CNTN6* gene variants.

## Footnotes

1 https://www.mousephenotype.org/ (accessed February 19, 2026).
